# Research progress on the role of bile acid metabolism in intestinal epithelial cell injury in necrotizing enterocolitis

**DOI:** 10.3389/fped.2026.1807109

**Published:** 2026-04-01

**Authors:** Sheng Zhang, Guijun Li, Yanjun Tian, Qi Liu

**Affiliations:** 1 Department of Pediatric Surgery, Peking University First Hospital Ningxia Women's and Children's Hospital, Yinchuan, Ningxia, China; 2 The Third Clinical Medical College of Ningxia Medical University, Yinchuan, Ningxia, China

**Keywords:** bile acids, Farnesoid X Receptor, ferroptosis, intestinal epithelial cell, intestinal microbiota, necrotizing enterocolitis

## Abstract

Necrotizing enterocolitis (NEC) is a critical gastrointestinal emergency frequently occurring in premature infants. Its etiology is not yet fully elucidated, which makes clinical diagnosis and treatment challenging. In recent years, the core role of bile acid metabolism dysregulation and the signaling pathways involved in the pathogenesis of NEC have become increasingly prominent. This review focuses on the core characteristics of abnormal bile acid composition and enterohepatic circulation imbalance in children with NEC, analyzing the bidirectional regulatory relationship between bile acids and intestinal microbiota. It also emphasizes the mechanism by which excessive activation of Farnesoid X receptor drives the occurrence and development of NEC by damaging the intestinal epithelial barrier, inducing ferroptosis in intestinal epithelial cells, and exacerbating intestinal immune inflammation. Intestinal epithelial cells are recognized as the central integrators and primary targets of bile acid dysregulation and intestinal microbiota dysbiosis in NEC pathogenesis. This review systematically summarizes relevant research progress and explores the potential value and clinical translational prospects of novel prevention and treatment strategies targeting bile acid metabolism and signaling pathways, providing theoretical support for optimizing NEC diagnosis and treatment, and improving the prognosis of premature infants.

## 1 Introduction

Necrotizing enterocolitis (NEC) is a leading cause of morbidity and mortality among premature infants, particularly among those with low birth weight. The disease is characterized by rapid progression characterized by intestinal mucosal inflammation, barrier disruption, and ischemic necrosis. Despite advances in clinical management, morbidity and mortality remain high. Survivors often face long-term complications such as neurodevelopmental impairment, short bowel syndrome, and growth failure. This substantial burden on families and healthcare systems partly results from the pathophysiological mechanisms that remain unclear.

In recent years, there has been a paradigm shift in our understanding of NEC, moving from a purely inflammatory model to a framework that incorporates metabolic dysregulation. Central to this paradigm shift is the dysregulation of bile acid metabolism, which is now recognized as a key upstream driver of NEC pathogenesis (1). In premature infants, factors such as hepatic immaturity, incomplete enterohepatic circulation, intestinal dysbiosis, and clinical interventions, including broad-spectrum antibiotics and total parenteral nutrition, contribute to abnormal bile acid profiles (1, 2). Bile acids serve not only as digestive surfactants but also as potent signaling molecules regulating intestinal inflammation, epithelial barrier function, and immune responses, primarily through receptors such as the Farnesoid X receptor (FXR) and Takeda G-protein coupled receptor 5 (TGR5) (1, 3).

The intestinal epithelial cell (IEC) serves as a critical interface for host-microbiota interactions. The IEC is responsible for nutrient absorption, mucin secretion, antimicrobial peptide secretion, antigen presentation, and constant self-renewal (4). The IEC is both the first line of defense and the primary site of injury in NEC. Disturbances in bile acid homeostasis and microbiota composition converge specifically to target IECs, thereby affecting IEC survival, functional integrity, and regenerative capacity. This targeting is primarily mediated by the nuclear receptor FXR, which is highly expressed in IECs and regulates gene networks governing proliferation, inflammation, and cell death. Targeted inhibition of FXR can alleviate NEC (3).

**Figure 1:**
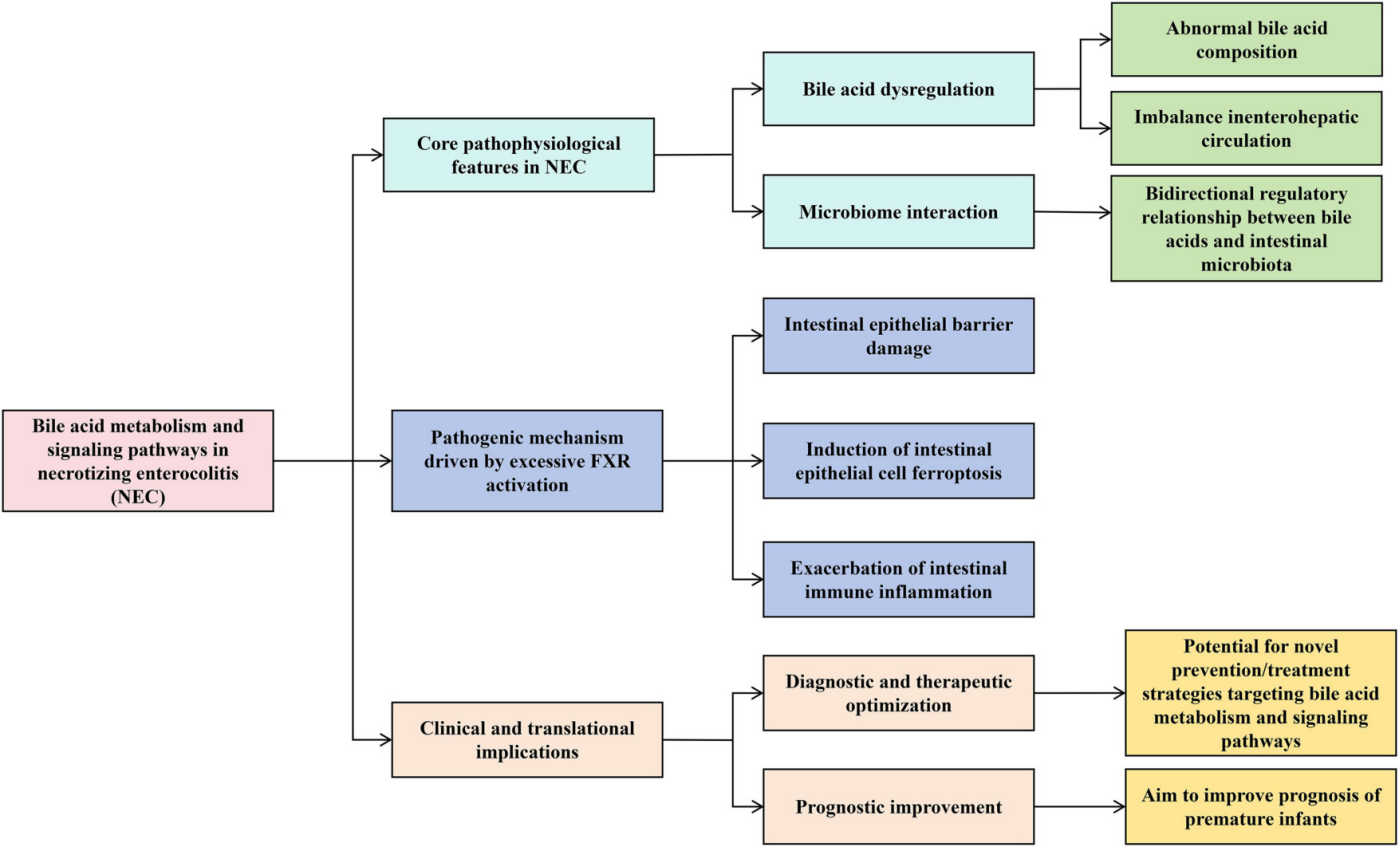
Bile acid metabolism and signaling pathways in necrotizing enterocolitis, showing core pathophysiological features, pathogenic mechanisms from excessive FXR activation, clinical implications, and resulting prospects for novel prevention, diagnosis, and prognosis in premature infants.

This review summarizes recent advances in bile acid metabolism and signaling in NEC, with a particular focus on the pivotal role of IECs (Figure 1). We explore the potential of mechanism-based prevention and treatment strategies, aiming to provide a theoretical framework that improves diagnosis and prognosis in premature infants.

## 2 Bile acid metabolism dysregulation and intestinal microbiota dysbiosis in NEC

The elevated risk of NEC is not attributable to a single defect; rather, it results from the immaturity and dysregulation occurring simultaneously in two closely interdependent systems: host bile acid metabolism and the commensal intestinal microbiota. This combined dysfunction creates an adverse luminal environment that directly disrupts the IEC homeostasis.

### 2.1 The dysregulation of bile acid metabolism and enterohepatic circulation in NEC

The preterm liver is functionally immature, as evidenced by an impaired and qualitatively altered bile acid synthesis profile. The activity of cholesterol 7α-hydroxylase (CYP7A1), the rate-limiting enzyme for the classic, neutral, pathway of bile acid synthesis, is significantly decreased (5). This phenomenon is persistent and sustained under conditions of physiological stress. Clinical observations of preterm infants requiring prolonged parenteral nutrition (PN) show consistently reduced CYP7A1 activity, which contrasts sharply with the normal age-dependent upregulation seen in healthier counterparts fed enterally (5). The mechanisms underlying this suppression are multifaceted. These mechanisms include the accumulation of phytosterols found in soybean oil-based lipid emulsions and, importantly, the abnormal activation of the fibroblast growth factor 19 (FGF19) signaling axis in the ileal hormone (5, 6). FGF19, produced by IECs upon FXR activation by bile acids, potently inhibits CYP7A1 transcription in the liver. In preterm infants, plasma FGF19 concentrations are significantly higher at birth than in term infants and then decline slowly. This contributes to a sustained physiological repression of bile acid synthesis, which may eventually progress to a pathological deficiency (6).

Paradoxically, a maladaptive alteration in bile acid reabsorption occurs in the microenvironment of the intestine affected by developing NEC, leading to detrimental cellular effects. The ileal apical sodium-dependent bile acid transporter (ASBT), which efficiently retrieves conjugated bile acids from the lumen into IECs, is markedly upregulated (1, 2). This upregulation, potentially driven by inflammatory cytokines or specific dysbiotic bacteria, such as Clostridium scindens (2), leads to the abnormal intracellular accumulation of bile acids, particularly hydrophobic subtypes that are intrinsically cytotoxic, such as deoxycholic acid. This accumulation triggers significant oxidative stress and endoplasmic reticulum stress within IECs, directly damaging cellular organelles and membrane structures (1). This accumulation also disrupts the normal feedback dynamics of FXR signaling, thereby promoting a pathological state (7). Genetically engineered mouse models demonstrate that ASBT is essential for physiological neonatal bile acid reabsorption and systemic homeostasis (8). However, the upregulation of ASBT during NEC creates a detrimental positive feedback loop, exacerbating epithelial injury.

### 2.2 Intestinal microbiota dysbiosis and its transformative impact on the bile acid pool

The intestinal microbiota functions as a virtual “secondary liver,” which plays an indispensable role in shaping the chemical landscape of bile acids through enzymes such as bile salt hydrolase (BSH) and 7α-dehydroxylase. These enzymes deconjugate and transform primary bile acids (cholic acid, chenodeoxycholic acid) into various secondary bile acids such as deoxycholic acid and lithocholic acid, which have distinct signaling and biological properties. The preterm intestinal microbiota is typically unstable, exhibiting low alpha-diversity and is vulnerable to disturbances caused by common perinatal factors such as cesarean delivery, formula feeding, and especially prophylactic or therapeutic antibiotic exposure (9). This state of dysbiosis results in a markedly diminished and qualitatively changed ability to transform bile acids. It is characterized by both a reduction in overall microbial biomass and a specific loss of beneficial bile acid-transforming bacterial taxa, such as members of the Clostridia clusters XIVa and IV. Additionally, a concomitant overgrowth of potentially pro-inflammatory species often occurs (10, 11).

Importantly, this host-microbe dialogue is inherently bidirectional and establishes a fundamental developmental interaction. Alterations in bile acid metabolism and intestinal microbiota, both closely associated with increased susceptibility to intestinal inflammation, have also been observed in newborns of mothers with intrahepatic cholestasis of pregnancy (12). Moreover, host-derived bile acids not only serve as substrates but also act as active, instructive signals that drive the assembly, succession, and maturation of the postnatal intestinal microbiota (13). Therefore, the deficiency and dysregulation inherent in bile acids of premature infants can directly impede the establishment of a healthy microbiota, thus initiating a vicious cycle from the outset. The immediate and profound functional consequences of established dysbiosis include significant alterations in microbial metabolism and host physiology. An increase in bacteria with high 7α-dehydroxylase activity, such as C. scindens, can shift the bile acid pool composition toward a higher proportion of hydrophobic secondary bile acids. These hydrophobic secondary bile acids, in turn, can further upregulate ASBT expression and enhance the absorption of these bile acids, leading to intracellular toxicity (2, 11). Furthermore, dysbiosis often leads to the depletion of key microbial metabolites, particularly the short-chain fatty acid butyrate (SCFA). Butyrate, produced by fermentative bacteria, acts as a histone deacetylase inhibitor and a partial antagonist of FXR signaling in IECs. The loss of butyrate during dysbiosis removes a crucial brake on FXR activity, thereby facilitating its pathological overactivation (3, 14).

The neonatal stage is a critical developmental phase marked by heightened metabolic vulnerability linked to the bile acid-microbiota-epithelium axis. Emerging research shows that the inherent metabolic immaturity of the newborn gut, together with the specific profile of bile acid metabolites in breast milk, significantly contributes to susceptibility to enteric inflammation and viral infections (15). This highlights how the “bile acid-microbiota-epithelium” axis operates within a critical developmental context. Physiological signaling is crucial for the proper development of the immune and barrier functions, and its absence or distortion predisposes individuals to diseases such as NEC.

## 3 Core mechanisms of bile acid-induced intestinal epithelial injury in NEC

The convergence of an abnormal, cytotoxic bile acid pool and a dysbiotic, butyrate-depleted luminal environment upon the vulnerable IEC monolayer activates specific, interconnected molecular pathways that drive the necro-inflammatory cascade of NEC.

### 3.1 Pathological FXR activation and the ferroptosis in IECs

FXR is a ligand-activated nuclear receptor that serves as the master regulator of bile acid homeostasis. It is abundantly expressed within the epithelium of the ileum and colon. In the context of NEC, increased ligand availability (accumulated bile acids) combined with the loss of endogenous inhibitors (butyrate) leads to pathological overactivation of FXR signaling. This is corroborated by clinical and preclinical evidence demonstrating significantly elevated FXR protein levels and target gene expression in the intestinal epithelium of NEC patients and experimental models. Moreover, FXR protein and target gene expression levels show a positive correlation with tissue damage severity (3, 14).

The excessive activation of FXR directly induces a novel and potent form of cell death in IECs: ferroptosis. Unlike apoptosis or necrosis, ferroptosis is an iron-dependent, regulated form of cell death distinct from apoptosis, driven by the extensive peroxidation of phospholipids containing polyunsaturated fatty acids (PUFAs). The mechanism involves activated FXR translocating to the nucleus and binding to specific response elements within the promoter region of the gene encoding Acyl-CoA synthetase 4 (ACSL4). ACSL4 is a key rate-limiting enzyme that catalyzes the esterification of free PUFAs, such as arachidonic acid and adrenic acid, into their CoA derivatives, facilitating their incorporation into membrane phospholipids, particularly phosphatidylethanolamines. By significantly increasing the content of peroxidation-susceptible PUFAs in cellular membranes, ACSL4 upregulation renders IECs highly susceptible to oxidative damage (3). In the inflammatory, iron-replete microenvironment of the NEC intestine, reactive oxygen species catalyze the peroxidation of these PUFA-rich membranes, resulting in the loss of membrane integrity, disruption of osmotic balance, and ultimately, ferroptotic cell death. Thus, the FXR-ACSL4-ferroptosis axis has been identified as a key mechanism driving the specific and widespread loss of IECs characteristic of NEC (3). Genetic ablation of intestinal FXR or pharmacological inhibition of either ACSL4 or ferroptosis significantly reduces epithelial damage and inflammation, and improves survival in murine NEC models (3).

The damage initiated by ferroptosis extends beyond the physical demise of the IECs. Lipid peroxide byproducts such as 4-hydroxynonenal and malondialdehyde released from ferroptotic cells significantly dysregulate the local innate immune landscape by acting as damage-associated molecular patterns (DAMPs). One critical dysfunction that occurs is the suppression of interleukin-22 (IL-22) secretion by group 3 innate lymphoid cells (ILC3s) residing in the intestinal lamina propria (3). IL-22 is a key cytokine that is essential for maintaining epithelial health. It acts directly on IECs by promoting stem cell proliferation, enhancing tight junction protein expression, and stimulating the production of antimicrobial peptides (16). The loss of IL-22 signaling impairs the intestine's innate capacity for rapid repair and effective chemical defense, thus establishing a vicious cycle. Epithelial damage initially caused by ferroptosis impairs immune support mechanisms. This loss of support exacerbates subsequent epithelial injury and facilitates bacterial translocation (17). This pathogenic interplay is reflected in the clinical setting, where elevated plasma lipid peroxidation products in NEC patients positively correlate with disease severity and FGF19, whereas circulating IL-22 shows an inverse correlation with these parameters (3).

### 3.2 The integrated microbiota-bile acid-epithelium signaling network

Changes in the microbial community structure can lead to increased consumption of short-chain fatty acids, thereby establishing a vicious cycle of disorders in bile acid metabolism (18). Antibiotics selectively inhibit microbiota that produce secondary bile acids, including Ruminococcaceae and Clostridiaceae. This inhibition blocks the conversion of primary bile acids to secondary bile acids, leading to an accumulation of primary bile acids and a reduction of secondary bile acids (19). Clinical studies demonstrate that ceftazidime treatment significantly decreases secondary bile acid levels while increasing primary bile acid levels in the gastrointestinal tracts of premature infants (20). Moreover, commonly used antibiotics, including moxifloxacin and metronidazole, significantly inhibit the activity of key intestinal microbiota responsible for secondary bile acid production (21). These changes may have variable effects on epithelial inflammation and barrier function, thereby directly altering the signaling environment of IEC.

Microbiota-derived secondary bile acids are not simply waste products, they also function as active immunomodulators (22). For instance, hyocholic acid, a microbially modified bile acid, enhances the production of IL-22 and other type 3 immune cytokines, thereby strengthening the epithelial barrier and protecting neonates against invasive pathogens of the enteric system, such as Salmonella Typhimurium (23). This exemplifies how a well-functioning microbiota-bile acid circuit provides essential metabolite-mediated support to the epithelium.

The interplay among the triad of bile acids, microbiota, and epithelial cells is both continuous and dynamic. Bile acids, through their inherent antimicrobial properties, exert selective pressure that shapes the composition of the microbiota. In return, microbiota-derived metabolites like SCFAs fine-tune the activity of bile acid receptors and influence epithelial gene expression. In NEC, this delicate homeostasis is disrupted. Dysbiosis, including ASBT-mediated intracellular bile acid accumulation and the loss of microbial metabolites such as butyrate collectively exert a multi-faceted impact on IECs. This assault is mediated by receptors such as FXR and TGR5. It compromises tight junction integrity, activates pro-inflammatory NF-κB pathways, induces specific forms of cell death, such as ferroptosis, and impairs epigenetically regulated programs essential for tissue repair and regeneration.

## 4 Diagnostics and therapeutics

The detailed mechanistic dissection of the bile acid-IEC axis transitions from explaining disease to offering tangible, novel pathways for clinical translation in NEC, spanning improved diagnostics and innovative therapeutics.

### 4.1 Emerging biomarkers: FGF19 and lipid peroxides

The current clinical diagnosis of NEC relies on a combination of non-specific clinical signs (abdominal distension, bloody stools) and radiographic findings (pneumatosis intestinalis), which typically manifest only after significant injury has taken place. Molecules directly involved in the core pathology, such as plasma FGF19 and lipid peroxidation markers, hold great promise as objective and quantifiable biomarkers. FGF19, which is a direct transcriptional target of FXR, serves as a systemic indicator of intestinal FXR activity. Clinical studies consistently show that FGF19 levels are significantly elevated in NEC patients relative to gestational age-matched healthy preterm controls. Moreover, higher concentrations correlate with more severe disease, as classified by Bell's stage, and predict an increased risk of mortality (3). Similarly, lipid peroxide levels, as indicated by malondialdehyde measurements or the ferrous oxidation-xylenol orange assay, are elevated in NEC serum and correlate with clinical severity. Importantly, these levels decrease in response to clinical improvement, suggesting their utility not only for diagnosis but also for monitoring therapeutic response (3). The distinct, positive correlation of FGF19 with NEC disease activity is particularly noteworthy, since this differs from its pattern in other inflammatory bowel diseases, such as Crohn's disease, potentially offering a disease-specific diagnostic signature (24, 25). Future work should focus on validating the sensitivity and specificity of these biomarkers and determining the optimal timing for sample collection in large, prospective multicenter cohorts.

### 4.2 Novel therapeutic strategies rooted in mechanism

Given the clearly central role of pathological FXR signaling in driving IEC ferroptosis, pharmacologically inhibiting this receptor specifically in the gut is a rational and promising strategy (26–28). Preclinical proof-of-concept studies demonstrate that orally administered, minimally absorbed FXR antagonists can locally antagonize ileal FXR, suppress ACSL4 upregulation, reduce markers of ferroptosis and inflammation, alleviate histological damage, and improve survival significantly in rodent NEC models (29, 30). Their local mode of action minimizes the risk of systemic side effects, a crucial consideration for vulnerable preterm infants, and enhances their potential for clinical translation.

Targeting the downstream executioner pathway of ferroptosis offers an additional or alternative intervention point. Pharmacological inhibitors of ferroptosis such as ferrostatin-1, liproxstatin-1, or specific ACSL4 inhibitors have demonstrated efficacy in protecting IECs against bile acid-induced cell death. These inhibitors also preserve barrier function, reduce intestinal damage, and ameliorate pathological outcomes in experimental NEC models (3). This strategy is attractive as it directly targets epithelial rescue, potentially halting disease progression even after the initial FXR activation signal has been transmitted.

This category includes indirect strategies aimed at reducing harmful signals that reach the epithelium. Bile acid sequestrants bind to luminal bile acids, reducing their reabsorption and their overall concentration in the intestinal lumen. This process lowers the agonist load on FXR and decreases direct cytotoxic stress (1). A more physiological approach involves restoring a healthier microbial community through the administration of targeted probiotic strains, prebiotics, or fecal microbiota transplantation (31). The goal is to normalize bile acid metabolism, increase the proportion of beneficial secondary bile acids that have anti-inflammatory or FXR-antagonizing properties, and restore the production of protective metabolites such as butyrate. For instance, specific probiotic formulations have been shown to alter the cecal bile acid profile in experimental models, increasing the ratio of FXR antagonists to agonists (32, 33).

Long-term PN is a major, independent risk factor for both NEC and PN-associated cholestasis, partly due to the previously described suppression of bile acid synthesis (34–36). Mitigation strategies should be multifaceted. They include optimizing PN formulations, such as the use of fish oil-based or multi-oil lipid emulsions to reduce phytosterol load; cautiously and diligently advancing enteral feeding even minimal volumes to stimulate biliary secretion and the enterohepatic circulation; and exploring the adjunctive use of hepatoprotective agents such as ursodeoxycholic acid or specific probiotics, including Lactobacillus plantarum, that may support bile acid metabolism and gut barrier function (37–39).

## 5 Conclusion

In conclusion, this review synthesizes the pathogenesis of NEC, emphasizing bile acid metabolism and its complex bidirectional interactions with the intestinal microbiota, which serve as core driving factors. IECs are recognized as the central integrators and primary targets of dysbiosis.

The preterm infant's distinctive physiological vulnerabilities, including an immature bile acid synthesis system and an unstable early pioneer microbiota, create a fragile luminal environment. This environment affects the IEC monolayer, predominantly through excessive and dysregulated FXR signaling. This pathological activation triggers a specific cell death program (ferroptosis) while simultaneously impairing the innate immune-mediated pathways essential for epithelial repair and barrier reinforcement.

Thus, the “bile acid-intestinal epithelial injury” axis offers a robust, coherent framework integrating previously unconnected clinical and experimental observations. It extends beyond correlation by elucidating causative mechanisms and most importantly highlights clear, actionable translational strategies, including the development of mechanism-based biomarkers and the rational design of novel therapeutic approaches.
